# Early cortical microstructural neurodegeneration in aging is linked to vulnerability to Alzheimer's disease pathology

**DOI:** 10.1002/alz.093632

**Published:** 2025-01-09

**Authors:** Rongxiang Tang, Carol E. Franz, Richard L. Hauger, Anders M. Dale, Stephen M Dorros, Lisa T. Eyler, Christine Fennema‐Notestine, Donald J. Hagler, Michael J. Lyons, Matthew S. Panizzon, Olivia K. Puckett, McKenna E. Williams, Jeremy A. Elman, William S. Kremen

**Affiliations:** ^1^ University of California, San Diego, La Jolla, CA USA; ^2^ Center for Behavior Genetics of Aging, School of Medicine, University of California, La Jolla, CA USA; ^3^ University of San Diego California, San Diego, CA USA; ^4^ Boston University, Boston, MA USA

## Abstract

**Background:**

Early identification of Alzheimer’s disease (AD) risk prior to irreversible brain damage is critical for improving the success of interventions and treatment. Cortical thickness is a macrostructural measure typically used to assess AD neurodegeneration. However, cortical microstructural changes appear to precede macrostructural atrophy and may improve early identification of AD risk. Currently, whether cortical microstructural neurodegeneration in aging is linked to early vulnerability to AD pathophysiology remains unclear in non‐clinical populations, who are precisely the target population for early risk identification.

**Method:**

In 194 dementia‐free community‐dwelling adults, we calculated MRI‐derived group‐level brain maps of longitudinal changes in cortical mean diffusivity (microstructure) and cortical thickness (macrostructure) over 5‐6 years (mean age: Time 1=61.82; Time 2=67.48). We obtained PET‐derived group‐level brain maps of stereotypical AD pathology deposition (beta‐amyloid and tau) and density maps of neurotransmitter receptors (cholinergic, glutamatergic) implicated in AD pathophysiology. An MRI‐derived map of cortical organization (sensorimotor‐association axis) was used to probe general age‐related changes. Spatial correlational analyses were used to compare pattern similarity among maps. We first examined whether changes in cortical microstructure and macrostructure are enriched in regions with increased accumulation of beta‐amyloid and tau, and higher density of cholinergic and glutamatergic receptors. We then investigated whether individuals with more AD‐like change profiles (spatially similar to beta‐amyloid or tau deposition patterns) showed greater memory decline as measured by three well‐established neuropsychological tests.

**Result:**

Spatial patterns of cortical macrostructural changes only resembled patterns of cortical organization sensitive to general age‐related processes (r=‐0.31, p<0.05), whereas microstructural changes resembled stereotypical patterns of beta‐amyloid (r=0.20, p=0.015) and tau (r=0.41, p=0.015) deposition in AD. Individuals with patterns of microstructural changes that more closely resembled stereotypical tau deposition exhibited greater memory decline (ß=0.21, p=0.036). Moreover, microstructural changes and AD pathology deposition were enriched in areas with greater densities of cholinergic and glutamatergic receptors (ps<0.05).

**Conclusion:**

Patterns of cortical microstructural neurodegeneration were more AD‐like than patterns of macrostructural neurodegeneration, which appeared to reflect more general aging processes. Microstructural changes may therefore better inform early risk prediction efforts as a sensitive measure of vulnerability to pathological processes prior to overt atrophy and cognitive decline.

